# Automated Nuclear Morphometry: A Deep Learning Approach for Prognostication in Canine Pulmonary Carcinoma to Enhance Reproducibility

**DOI:** 10.3390/vetsci11060278

**Published:** 2024-06-17

**Authors:** Imaine Glahn, Andreas Haghofer, Taryn A. Donovan, Brigitte Degasperi, Alexander Bartel, Theresa Kreilmeier-Berger, Philip S. Hyndman, Hannah Janout, Charles-Antoine Assenmacher, Florian Bartenschlager, Pompei Bolfa, Michael J. Dark, Andrea Klang, Robert Klopfleisch, Sophie Merz, Barbara Richter, F. Yvonne Schulman, Jonathan Ganz, Josef Scharinger, Marc Aubreville, Stephan M. Winkler, Christof A. Bertram

**Affiliations:** 1 Institute of Pathology, University of Veterinary Medicine Vienna, 1210 Vienna, Austria; 2 Bioinformatics Research Group, University of Applied Sciences Upper Austria, 4232 Hagenberg, Austria; 3 Department of Computer Science, Johannes Kepler University, 4040 Linz, Austria; 4 Department of Anatomic Pathology, The Schwarzman Animal Medical Center, New York, NY 10065, USA; 5 University Clinic for Small Animals, University of Veterinary Medicine Vienna, 1210 Vienna, Austria; 6 Institute for Veterinary Epidemiology and Biostatistics, Freie Universität Berlin, 14163 Berlin, Germany; 7 Comparative Pathology Core, Department of Pathobiology, University of Pennsylvania, Philadelphia, PA 19104, USA; 8 Institute of Veterinary Pathology, Freie Universität Berlin, 14163 Berlin, Germany; 9 Department of Biomedical Sciences, Ross University School of Veterinary Medicine, Basseterre P.O. Box 334, Saint Kitts and Nevis; 10 College of Veterinary Medicine, University of Florida, Gainesville, FL 32611, USA; 11 IDEXX Vet Med Labor GmbH, 70806 Kornwestheim, Germany; 12 Antech Diagnostics, Mars Petcare Science and Diagnostics, Fountain Valley, CA 92708, USA; 13 Department of Computer Science, Technische Hochschule Ingolstadt, 85049 Ingolstadt, Germany

**Keywords:** anisokaryosis, artificial intelligence, dog, image processing, mitotic count, nuclear pleomorphism, prognosis, pulmonary carcinoma

## Abstract

**Simple Summary:**

We investigated a new method for diagnosing and predicting outcomes in canine pulmonary carcinoma. We developed a deep learning-based algorithm that accurately detects tumor nuclei and subsequently measures size and shape parameters. The variation in nuclear size and shape (nuclear pleomorphism) is a crucial malignancy criterion used in the current grading system for canine pulmonary carcinoma. Pathologists currently evaluate it and classify it according to a three-tier system. Manual morphometry is a more objective approach where tumor nuclei are individually encircled and analyzed. This task can be easily performed by an algorithm. Our algorithm’s accuracy in correctly detecting and segmenting tumor nuclei was considered good when compared to manual morphometry. By comparing automated morphometry with conventional prognostic tests, such as pathologists’ estimates, mitotic count, histological grading, and clinical staging, we found that our approach was equally accurate in terms of prognostic value. The algorithm’s advantage lies in its high reproducibility and efficiency. Automated evaluation of nuclear pleomorphism can enhance the efficiency and reliability of canine pulmonary carcinoma diagnosis and grading, effectively addressing issues of inter-observer reproducibility. However, further optimization of the algorithm and validation with a larger study group is necessary to confirm our findings.

**Abstract:**

The integration of deep learning-based tools into diagnostic workflows is increasingly prevalent due to their efficiency and reproducibility in various settings. We investigated the utility of automated nuclear morphometry for assessing nuclear pleomorphism (NP), a criterion of malignancy in the current grading system in canine pulmonary carcinoma (cPC), and its prognostic implications. We developed a deep learning-based algorithm for evaluating NP (variation in size, i.e., anisokaryosis and/or shape) using a segmentation model. Its performance was evaluated on 46 cPC cases with comprehensive follow-up data regarding its accuracy in nuclear segmentation and its prognostic ability. Its assessment of NP was compared to manual morphometry and established prognostic tests (pathologists’ NP estimates (*n* = 11), mitotic count, histological grading, and TNM-stage). The standard deviation (SD) of the nuclear area, indicative of anisokaryosis, exhibited good discriminatory ability for tumor-specific survival, with an area under the curve (AUC) of 0.80 and a hazard ratio (HR) of 3.38. The algorithm achieved values comparable to manual morphometry. In contrast, the pathologists’ estimates of anisokaryosis resulted in HR values ranging from 0.86 to 34.8, with slight inter-observer reproducibility (k = 0.204). Other conventional tests had no significant prognostic value in our study cohort. Fully automated morphometry promises a time-efficient and reproducible assessment of NP with a high prognostic value. Further refinement of the algorithm, particularly to address undersegmentation, and application to a larger study population are required.

## 1. Introduction

Primary lung tumors in dogs are rare, although recognized cases have increased over the past two decades [1,2], occurring with a lifetime incidence of up to 8.8% [3]. Among these tumors, canine pulmonary carcinomas (cPCs) are the most prevalent [3,4,5,6,7,8,9,10]. The most common histologic type is invasive adenocarcinoma [4,5,9]. Metastasis occurs in 17–38% of the cases by hematogenous, lymphatic, or airway routes, mainly to the tributary (tracheobronchial) lymph nodes, other lung locations, and pleura/mediastinum [2,6,11,12,13].

The known relevant criteria for prognostic evaluation of cPC cases include tumor-node-metastasis (TNM) staging [7,10,11,14], mitotic count [4,7,15], and histological grade [7,14,16,17]. A high mitotic count (defined by previous studies as the number of mitotic figures per 10 high-power fields, without a defined area) as a solitary parameter has been associated with shorter survival and disease progression in cPC [7,15]. The median survival time of dogs with pulmonary carcinomas has been correlated with the histological grade in two studies. The first study by McNiel et al. [7] revealed median survival times ranging from 5 days for grade 3 tumors to 790 days for grade 1 tumors. The second study by Lee et al. [14] showed a range of median survival times from 43 days in grade 3 tumors to 658 days in grade 1 tumors [7,14]. Within the grading system of canine lung tumors, the overall tumor differentiation, number of mitotic figures, and nuclear pleomorphism have been shown to have the highest association with outcome, followed by nucleolar size, fibrosis, and degree of invasion [7].

Nuclear pleomorphism, which describes the variation in nuclear size and/or shape, is recognized as a prognostically relevant histological criterion in cPC (as a solitary parameter and as part of the grading system) and in various other tumor types [7,18,19,20,21].

The current approach for assessing nuclear pleomorphism in cPC, following the methods of the 1997 grading system [7], is the categorical estimation into three classes based on the variation in nuclear size (anisokaryosis) and shape irregularity. Nuclear pleomorphism estimates by pathologists are considered to be limited by low reproducibility, as has been shown for canine mammary carcinoma [22,23] and canine cutaneous mast cell tumors [24] and is suspected for cPC.

To improve the degree of reproducibility, alternative approaches for the assessment of nuclear pleomorphism are of interest. Computerized measurements can be performed either by pathologists using measurement software (manual morphometry) [23] or by the use of image analysis algorithms (automated/algorithmic morphometry) [25,26]. While manual morphometry performed by pathologists is too time-consuming for a routine diagnostic setting [23,27], automated morphometry and deep learning-based algorithms are efficient, given that an algorithm can segment and measure large numbers of nuclei with comparatively little computing power in a short period of time. The application of automated image analysis is becoming increasingly of interest for diagnostic laboratories that have implemented digital microscopy in their workflow [28].

The objective of this study was to investigate the reproducibility of nuclear pleomorphism estimates and the feasibility of automated nuclear morphometry for the prognostic evaluation of cPC. We hypothesized that automated morphometry would be equivalent to manual morphometry in terms of prognostic validity, whereas pathologists’ estimates are hampered by insufficient inter-observer reproducibility.

We had the following hypotheses:

1. Inter-observer reproducibility of nuclear pleomorphism estimates between pathologists is low in cPC, justifying the investigation of nuclear morphometry.
2. Algorithmic morphometry is able to accurately measure nuclear size and shape parameters.
3. Algorithmic morphometry is at least equivalent to manual morphometry nuclear pleomorphism estimates (which is impractical for a routine diagnostic test) and other established prognostic tests with regard to prognostic ability.

## 2. Materials and Methods

### 2.1. Material (Study Cases and Datasets)

The cPC cases were obtained from the diagnostic archives of veterinary pathology laboratories from two institutions (The Schwarzmann Animal Medical Center New York (AMC) and University of Veterinary Medicine Vienna (VMU)), which were separated into the ground truth dataset and outcome dataset (see below). Histological sections were created from representative paraffin blocks and stained with hematoxylin and eosin. Digitization of the histological sections was performed with the Pannoramics Scan II whole-slide image (WSI) scanner (3DHistech, Budapest, Hungary) at default settings with a scan magnification of 400× (resolution of 0.25 µm/pixel).

The selection of three (outcome dataset) regions or one (ground truth dataset) region of interest (ROI) within each WSI was performed using the open-source viewing and annotation software SlideRunner (Version 2.0.0) [29]. The higher number of the ROI for the outcome cases was chosen to account for possible tumor heterogeneity during prognostic evaluation. Each ROI had a size of 0.1185 mm^2^, which is equivalent to a 0.5 standard high-power field (HPF) according to the definition of Meuten et al. [28] and an aspect ratio of 4:3. The ROI selection was performed at low magnification intending to encompass a representative tumor region without specific emphasis on nuclear characteristics. The areas with necrosis, severe inflammation, large cystic spaces, and/or poor tissue preservation were excluded, if possible. Each ROI was cropped and exported from the WSIs as TIFF files using lossless compression.

#### 2.1.1. Outcome Dataset

The outcome dataset was used to evaluate the inter-observer reproducibility of the nuclear pleomorphism estimates and the prognostic value of the different tests (see below). This dataset consisted of 19 cases from the AMC and 27 cases from VMU (total *n* = 46), for which patient follow-up was available through the clinical records of the respective institutes. The outcome information of the AMC cases was available through a previous study currently under review (unpublished data) [30].

All the cases of cPC submitted for histological examination by the university clinic for small animals at VMU between April 2001 and 2021, with available follow-up information, were included in this study. The clinical records of the included cases were screened for the following information: (1) date of surgery, (2) date of death or loss to follow-up, (3) suspected cause of death based on clinical findings, (4) treatment regimens, and (5) patient signalment (breed, age, and sex).

#### 2.1.2. Ground Truth Dataset

For the modeling and testing of the developed algorithm, a ground truth dataset was created. A total of 40 cases were selected from VMU, and 3 cases were chosen from the AMC. For these 43 cases, the patient follow-up information was not available, and, thus, these cases were not eligible for the outcome dataset. Consequently, these cases were used for the development of the algorithm. The images were annotated using the software SlideRunner (Version 2.0.0) [29]. Each nucleus of the neoplastic epithelial cells was precisely outlined using the polygon annotation tool, resulting in 27,138 ground truth annotations (average per ROI: 631.12, range: 295–1224). To avoid inter-observer variability, this procedure was performed by a single annotator (IG) who was supervised by a board-certified pathologist (CAB). The images were randomly allocated to three subsets for algorithm training (*n* = 30), validation (*n* = 6), and testing (*n* = 7), while the cases within each subset varied for the three models (see below).

### 2.2. Methods

**Figure 1:**
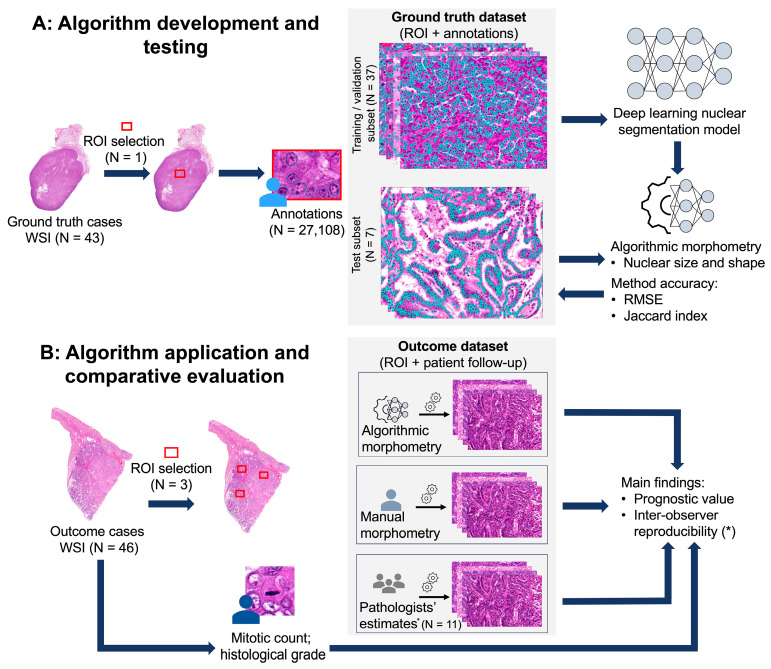
An overview of the development of the deep learning-based nuclear morphometry algorithm (**A**) and evaluation of its prognostic value (**B**). (**A**) The ground truth dataset, comprising 46 whole-slide images (WSIs) of canine pulmonary carcinoma (cPC) and the associated annotations, was utilized for the development, and testing of a deep learning-based algorithm for nuclear morphometry and the segmentation performance. (**B**) Independent cases with known patient survival (outcome dataset) were analyzed using this algorithm. Additional prognostic tests, including manual morphometry, pathologists’ estimates of nuclear pleomorphism, mitotic count, histological grade, and clinical stage, were performed on this dataset for comparison. *, The inter-observer reproducibility was evaluated and compared to the estimates provided by pathologists. ROI, region of interest; RMSE, root mean squared error; and WSI, whole slide image.

The deep learning-based algorithm for nuclear morphometry was developed with and tested against a ground truth dataset to evaluate its accuracy. Its prognostic value was evaluated on cases with a known patient survival time (outcome dataset). As a benchmark for the algorithm’s prognostic value, we compared it with manual morphometry, the pathologists’ estimates of nuclear pleomorphism, and additional prognostic tests (Figure 1). The inter-observer reproducibility of the nuclear pleomorphism estimates by the pathologists was assessed on the outcome dataset.

#### 2.2.1. Pathologists’ Estimates of Nuclear Pleomorphism

Eleven veterinary pathologists from 8 different laboratories evaluated the images of the outcome dataset (ROI 1) in a blinded manner with regard to the patient outcome and the results of the other pathologists and morphometry. For the data analysis, the participants were anonymized by randomly assigning a unique identification number (P1–P11). The pathologists applied one definition for anisokaryosis and shape irregularity each, which were modified from the grading system [7]. As compared to the previous definition, we removed anisocytosis (variation in cellular size) and divided nuclear pleomorphism into anisokaryosis (i.e., variation in nuclear size) and nuclear shape irregularity to allow for a better comparison with morphometric parameters, with the SD of the area reflecting anisokaryosis and the SD of the solidity the variation in shape.

The three anisokaryosis categories were defined as follows:

- Mild: an overall uniform nuclear size.
- Moderate: some variation in the nuclear size but with less than a two-fold difference.
- High: variation in the nuclear size with a greater than two-fold difference.

The two shape irregularity categories were defined as:

- Absent: regular (all or most round-to-oval nuclei with smooth contours).
- Present: irregular (numerous nuclei with uneven contours).

#### 2.2.2. Supervised Deep Learning-Based Algorithm (Fully Automated Morphometry)

We trained a deep learning segmentation model to detect individual tumor nuclei, using data augmentation techniques, followed by a morphometric analysis.

##### Deep Learning Segmentation Model

To detect the individual tumor nuclei, we combined semantic segmentation, i.e., the classification of each pixel, with connected-component labeling provided by the Scikit-Image framework [31]. As the segmentation model, we used a UNet++-based model [32] with a RegNetY120 [33] backbone provided by the segmentation model package [34] for Pytorch (Version 1.12.1) [35]. To be trained successfully, our model needed images and corresponding image masks as labels. Each labeled image must provide a binary mask with all the pixels that are part of the nuclei represented as the foreground (ones) and all the rest marked as the background (zeros). For the training of our model, we used the Pytorch Lightning framework (Version 1.7.2) [36] in combination with an adapted version of the focal loss [37]. This adaptation enabled the use of a weight map [38] that supported the model to learn how to provide a better separation of the individual nuclei. To assess the generalization performance of the employed model architecture, three models were trained on three different data splits, all separated into a training, validation, and test set, while ensuring no overlap between the test sets, always represented by 7 images. The 3 models were each trained for 2000 epochs using the training dataset. For each data split, the best model out of these epochs was selected based on the validation loss.

In order to select the optimal model based on validation performance, we employed the model checkpoint method of the Pytorch Lightning framework (Version 1.7.2) [36]. For monitoring the loss during the training process and saving example images and the corresponding segmentation masks for each individual epoch, we used Tensorboard [39] throughout the modeling process. This monitoring ensured optimal conditions for the model training and model selection process. To artificially increase the variation within our dataset, we applied data augmentation methods during the training steps using a randomly selected combination of methods, including transpose, vertical/horizontal flip, rotation, elastic transformation, grid/optical distortion, image shifting, image scaling, RGB color-channel shifts, and changes in brightness and contrast as well as changes in hue and saturation, as is commonly performed.

In addition to these augmentation methods, we used random cropping to extend our dataset artificially. Using a random selection of the regions and images to be used for the creation of these crops, we artificially extended our dataset by a factor of 10. For the validation dataset, the random cropping was performed once prior to training the model. For the training dataset, new random crops were taken after each epoch. Using the distributed data parallel (DDP) strategy of the Pytorch Lightning framework (Version 1.7.2) [36], we trained our models on two GPUs, each processing a batch of 8 image crops during each step of each epoch, resulting in a combined batch size of 16 crops per epoch step.

##### Algorithmic Morphometry

After the segmentation and localization of the individual nuclei and the filtering of objects with an area of <7 µm^2^, which were considered too small to be nuclei (i.e., “noise”), different morphometric parameters (Table 1) were calculated using the Scikit-Image framework [31]. The selection of these parameters is based on a previous study [40]. The nuclear area was defined by the pixel number within the segmented objects and subsequent conversion to µm² based on the scan resolution. The standard deviation (SD) of the nuclear area reflects the variation in the nuclear size and was therefore used as the primary parameter for comparison with the pathologists’ anisokaryosis estimates (see below). The percentage of large (karyomegalic) nuclei was determined by two nuclear size thresholds (42.3 and 50.5 µm^2^), which represent the 90th percentile and twice the median of the area of all the nuclei labeled in the ground truth dataset.

As indicators of the nuclear shape, we assessed the eccentricity and solidity using the Scikit-Image framework [31]. Eccentricity describes the roundness of the object and is determined by the ratio of the distance between the focal points of an ellipse and the length of its major axis. A ratio nearing 1 signifies a more elongated shape, while 0 indicates the perfect circularity of the shape.

To calculate the solidity of each segmented object, we employed the ratio of the object’s detected area relative to the area of its convex hull. A solidity value of 1 indicates perfect shape regularity. The closer this value is to 0, the more indentations are present and/or the larger the indentations are. Thus, the standard deviation of solidity is a reliable indicator for irregularity in the nuclear shape. To evaluate the prognostic significance of the mean and median solidity, we compared the values to the shape irregularity estimates conducted by the pathologists (see below). To ensure clarity, we inverted the values by calculating 1—the mean/median solidity value for each case—so that larger values represent increased shape irregularity.

The final algorithm was deployed to process the images of the test subset of the ground truth dataset and outcome dataset.

#### 2.2.3. Benchmark Prognostic Tests

To comprehensively evaluate the prognostic value of the algorithm, we compared its performance with the nuclear pleomorphism estimates by the pathologists, manual morphometry, and established prognostic tests including the mitotic count, histological grade, and clinical stage, using the outcome dataset (Figure 1).

##### Manual Morphometry

The manual morphometry was conducted by one author (IG) by annotating at least 100 tumor nuclei per image. An overlay featuring a 5 × 6 grid of thin black lines was added to the TIFF files, dividing the ROI images into 30 equally sized parts. With the aid of the annotation software SlideRunner (Version 2.0.0) [29], the annotator circled all the nuclei in as many grids as required until 100 nuclei were labeled. After reaching 100 nuclei, the current grid was completed, resulting in up to 137 (range 101–137 and mean = 115.91) annotations per image. The nuclei that were cut at the margins of the image were excluded, while the nuclei that intersected the grid lines were completely encircled (i.e., the annotation extended to the neighboring grid). The annotations for each grid started from the upper left corner and proceeded in a meandering pattern. The annotations were subsequently measured using the same morphometric parameters as for the algorithmic morphometry (Table 1).

##### Mitotic Count (MC)

For the MC, an ROI with the size of 2.37 mm^2^ at an aspect ratio of 4:3 was selected (bounding box annotation) by one pathologist (CAB) using the software SlideRunner (Version 2.0.0) [29], as previously described [41], in a tumor area with high mitotic activity. The areas with marked necrosis, inflammation, poor tissue preservation, and low cellular density were excluded from consideration, consistent with the current guidelines [28]. The area selection for the MC was independent from the area selection for the nuclear morphometry. Within these regions, two pathologists (CAB and TAD) independently annotated all the mitotic figures based on published morphologic criteria [42]. The number of mitotic figures per ROI was used as the MC value, while the MCs of both pathologists were used separately for the prognostic evaluation.

##### Histological Grade

The grade according to the methods described by McNiel et al. [7] (Supplementary Table S11) was determined in the WSI of the outcome cases. Initially, the cases were evaluated independently by CAB and TAD, and the cases with disagreement in the assigned grades were reviewed jointly for deriving consensus. The MC as determined above by the respective pathologist was used for grading.

##### Clinical Staging System

The clinical stage of the outcome cases was determined from the medical records based on the system developed by Owen et al. [43]. The stage is based on primary tumor features (T0–T3), regional lymph nodes (N0–N2), and distant metastasis (M0 and M1) and is classified in four tiers, with stage 1 suggesting a favorable and stage 4 a poor patient outcome.

#### 2.2.4. Statistical Analysis

GraphPad Prism version 5.0 (GraphPad Software, San Diego, CA, USA), IBM SPSS Statistics version 29.0 (IBM Corporation, Armonk, NY, USA), and R version 4.2.2 (R Foundation, Vienna, Austria) were used for the statistical analysis.

##### Inter-Observer Reproducibility

The inter-observer reproducibility between the estimates of the study participants on the outcome dataset was determined by Light’s Kappa (k) with an interpretation of the k-values as follows: 0 = poor agreement, 0.01–0.20 = slight agreement, 0.21–0.40 = fair agreement, 0.41–0.60 = moderate agreement, 0.61–0.80 = substantial agreement, and 0.81–1.00 = almost perfect agreement [44]. Linear regression was used to determine the correlation between the pathologists’ anisokaryosis estimates with the algorithmically measured SD of the nuclear area with spline regression for the smoothing of the resulting curves.

##### Test Accuracy of Algorithmic Morphometry

The test accuracy was determined on 21 images of the ground truth test subsets using cross validation of three models. Each of the three models used 7 test images, excluding any augmentation methods or image crops. Firstly, the model’s binary segmentation quality was determined through the Dice Coefficient. Secondly, we validated the localization performance of our model in combination with connected-component labeling. Conversely, we conducted a test to determine if all the nuclei identified in the labeled mask were included in the segmentation result and also to detect objects falsely segmented as nuclei. The F1 score, precision, and recall were used as quality measurements for the localization performance.

The root mean squared error (RMSE) was calculated for the whole image, comparing the difference between the algorithmic and manual ground truth measurements. Additionally, the RMSE-to-range ratio was calculated to describe the extent of the error in comparison to the distribution of the data.

##### Prognostic Value

The prognostic value of the nuclear evaluation methods and other prognostic indicators was determined based on the outcome dataset. For this, the tumor-specific survival time (time between primary surgery and death related to the cPC) and overall survival time (time between primary surgery and death related to any cause) were assessed. Based on the available patient follow-up information, 250-day survival was selected as a trade-off between a sufficiently long follow-up period and the need to exclude or censor cases with short follow-up. While exclusion/censoring affected 11 patients for the tumor-specific survival interval of 250 days, 21 patients would have been affected for the interval of 365 days. The patients that died of tumor-unrelated death or were lost to follow-up during the 250-day observation period were censored or excluded, respectively, from the analysis.

The prognostic value was evaluated using numerical and categorical values. The numerical tests (morphometry and MC) were analyzed by the area under the receiver operating characteristic curves (AUC), univariate cox regression (hazard ratios with 95% confidence intervals, 95% CI), and scatter plots (comparing cases with and without tumor-specific mortality).

The categorical tests (anisokaryosis and nuclear irregularity estimates, tumor grade, and stage) and dichotomized numerical tests were analyzed by sensitivity (Sen), specificity (Sp), precision (Pre), Kaplan–Meier curves, and univariate cox regression (hazard ratios with 95% CI). Dichotomization of the numerical tests was performed using scatter plots by selecting the threshold value resulting in a Sen of 70% (i.e., correct classification of 7/10 cases with tumor-related mortality) and the highest possible Sp value. The uniform Sen values allow for a comparison of the Sp values between the different prognostic tests. For the mitotic count, we applied the three established thresholds of the grading system for case categorization; a combination of these categories (1 and 2 vs. 3, and 1 vs. 2 and 3) was used for the statistical analysis [7].

## 3. Results

### 3.1. Inter-Observer Reproducibility of Nuclear Pleomorphism Estimates

**Figure 2:**
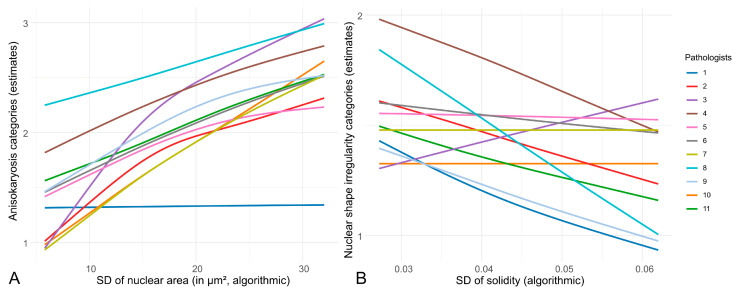
Linear regression curves of the individual pathologists’ three-tier anisokaryosis (**A**) and two-tier shape irregularity (**B**) estimates with the algorithmically measured standard deviation (SD) of the nuclear area and SD of the solidity, respectively. The curves show which anisokaryosis or shape irregularity score would likely be assigned by the respective pathologists depending on the SD of the nuclear size. The figure highlights the variability in the pathologists’ assessments, with different patterns in assigning in manual estimates, emphasizing the need for objective, algorithmic measures for consistent evaluation.

The inter-observer reproducibility was slight (k = 0.204) for the three-tier anisokaryosis estimates and fair (k = 0.272) for the two-tier nuclear shape irregularity. Figure 2 shows the difference between the individual pathologists in assigning an anisokaryosis or shape irregularity category depending on the algorithmically measured SD of the nuclear area and SD of the solidity.

### 3.2. Test Accuracy of Algorithmic Morphometry

**Figure 3:**
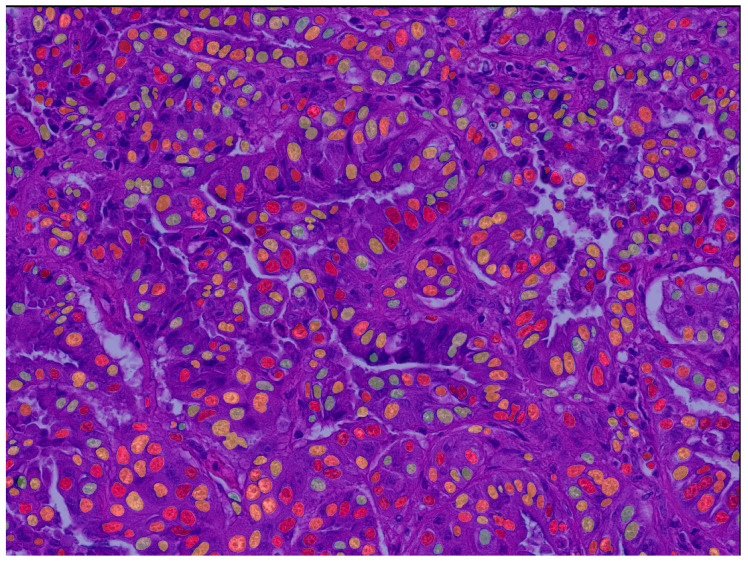
An exemplary image of the algorithmic segmentation showing the nuclei detection performance of model 1 in combination with the connected-component labeling. Each nucleus is colored randomly to get an impression for the model’s capability to separate the individual nuclei.

As listed in Table 2, the three segmentation models provide Dice scores (segmentation performance) above 0.7661 and F1-scores (object detection performance) above 0.8397 when combined with the connected-component labeling for detecting the individual nuclei (Figure 3 and Supplementary Figure S1). The root mean squared error (RMSE) was considered acceptable for the majority of the measured morphometric parameters (Supplementary Tables S1 and S2), while the RMSE values vary somewhat across the three models/test subsets and parameters. Notably, the parameters of the nuclear shape, particularly the SD of the solidity (range between the three test sets: 51–155%), show an overall larger RMSE-to-range coefficient compared to the features of the nuclear size, such as the SD of the area (range: 7.8–46.6%). Supplementary Figure S2 illustrates the positive linear correlation between the algorithmic and ground truth measurements for the selected morphometric parameters, combining the test cases for the three segmentation models.

### 3.3. Prognostic Value

The demographic characteristics of the study population are presented in the Supplementary Materials. The follow-up period ranged from 1 to 1961 days, with a median survival time or lost-to-follow-up time of 257 days. For the 15 cases, where death was attributed to cPC, the median survival time was 151 days. At 250 days after surgery, 8 dogs were lost to follow-up, 10 died of suspected tumor-related cause, 3 died of tumor-unrelated cause, and 25 were alive. A total of 19 dogs received chemotherapy in addition to surgical therapy. The clinical staging at the time of surgery identified 35 cases of stage 1, 5 cases of stage 2, and 3 cases each of stages 3 and 4. Metastases at death were confirmed by histology or cytology in 6 cases and suspected based on diagnostic imaging in 12 cases. The histological grading revealed that 6 dogs were classified as grade 1, 32 as grade 2, and 3 as grade 3. The mitotic figures were counted as mitoses per 10 high-power fields (HPFs) and scored according to the grading scheme proposed by McNiel et al. [7]. Pathologist 1 identified 23 dogs with a score of 1, 9 with a score of 2, and 7 each with scores of 3 and 4. Pathologist 2’s assessment categorized 14 dogs with a score of 1, 11 with a score of 2, 6 with a score of 3, and 15 with a score of 4.

**Figure 4:**
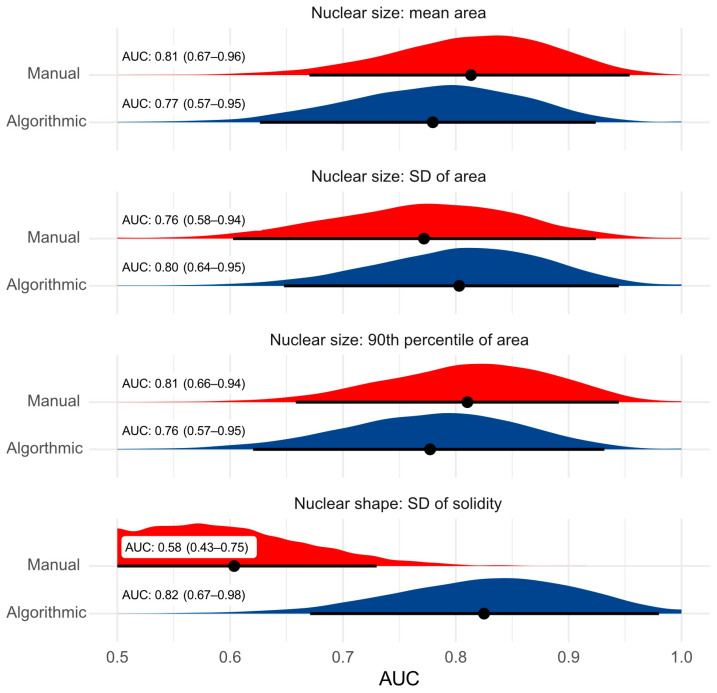
Graphical presentation of the area under the ROC curve values (black dots, tumor-specific survival at 250 days after surgery) and their 95% confidence intervals (black line) and probability density function (blue and red area) comparing algorithmic (blue, model 1 deployed on ROI 1) and manual nuclear morphometry (red). The analysis is based on 10 cases with tumor-specific mortality within the first 250 days after surgery and 25 cases that survived the follow-up period. AUC, area under the curve; SD, standard deviation.

While the MC of both pathologists (AUC_1_ = 0.474, 95%CI: 0.237–0.711; AUC_2_ = 0.374, 95%CI: 0.109–0.639) was unable to discriminate tumor-specific survival at 250 days, the algorithmic and manual nuclear morphometry achieved AUC values of above 0.75 for the nuclear area parameters, excepting skewness (Figure 4 and Supplementary Table S3). The SD of the solidity measurements resulted in the highest AUC values of 0.82 (95%CI: 0.67–0.98) for the algorithmic morphometry; however, the SD of the solidity measurements assessed manually was not prognostically relevant (Figure 4). The AUC values for all-cause mortality are provided in Supplementary Table S4. Analyzing the nuclear parameters in multiple tumor areas (one ROI vs. three ROIs) did not improve the prognostic ability (Supplementary Figure S3 and Tables S3 and S5).

**Figure 5:**
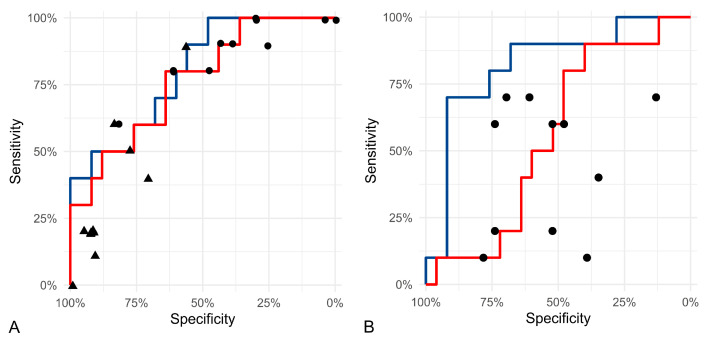
The ROC curves for the algorithmic (blue, model 1) and manual (red) morphometry and sensitivity/specificity values for the pathologists’ estimates (symbols) from ROI 1. (**A**) The curves represent the SD of the nuclear area measurements and the symbols the three-tier anisokaryosis estimates (dots: grade 1 + 2 vs. 3; triangle: grade 1 vs. 2 + 3). (**B**) The curves represent the SD of the solidity measurements and the dots the two-tier shape irregularity estimates by the pathologists.

The scatter plots for the algorithmic and manual morphometric measurements comparing cases with and without tumor-related death are shown in Supplementary Figure S4. The prognostic thresholds and the corresponding Sen and Sp values for the selected morphometric parameters are listed in Table 3. The categorical anisokaryosis estimates of the 11 pathologists show similar performance compared to the algorithmic and manual morphometry of the SD of the nuclear area but with highly variable Sen/Sp values ranging from Sen = 7.1%/Sp = 91.7% (pathologist 7) to Sen = 78.6%/Sp = 58.3% (pathologist 8) for anisokaryosis 1 and 2 vs. 3 and from Sen = 64.3%/Sp 50% (pathologist 2) to Sen = 100%/Sp = 0.0% (pathologist 8) for anisokaryosis 1 vs. 2 and 3 (Figure 5 and Supplementary Table S7). The algorithmic measurement of the SD of the solidity outperformed the manual morphometry and estimates by all 11 pathologists, which had highly variable performance ranging from Sen = 21.4%/Sp = 83.3% (pathologist 1) to Sen = 71.4%/Sp 12.5% (pathologist 4; Figure 5 and Supplementary Table S8). Supplementary Table S10 shows that other prognostic tests, such as the MC, grade, and stage, had highly variable Sen/Sp values, with precisions ranging from 18.8% to only up to 50.0%.

**Figure 6:**
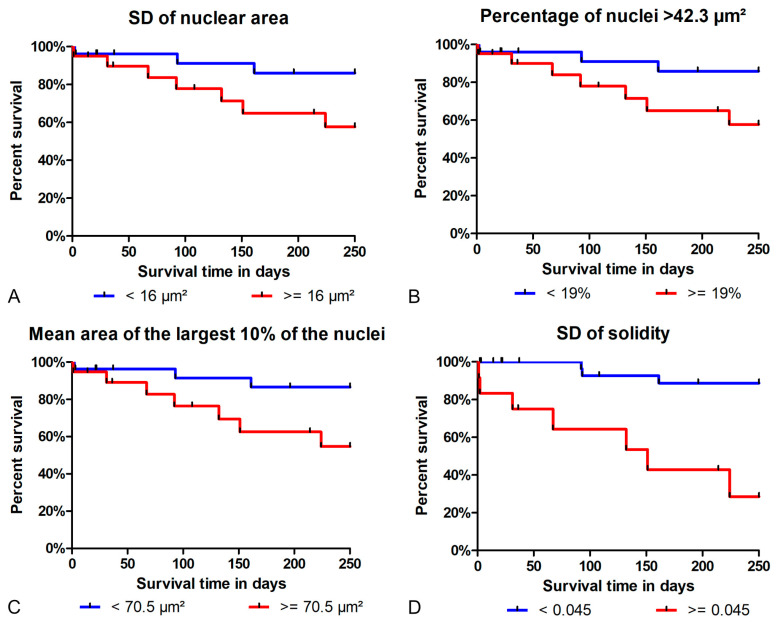
The Kaplan–Meier curves for the tumor-specific survival time (until 250 days after surgery) for the different nuclear size and shape parameters of the algorithmic morphometry (model 1, ROI 1). The analysis is based on 46 cases with the censoring of the cases that were lost to follow-up or died due to tumor-unrelated causes (*n* = 11). A total of 10 cases died of tumor-related causes and 25 dogs survived the follow-up period. (**A**) The standard deviation (SD) of the nuclear area (log rank test: *p* = 0.0615). (**B**) The percentage of nuclei > 42.3 µm^2^ (log rank test: *p* = 0.0658). (**C**) The mean area of the largest 10% of the nuclei (log rank test: *p* = 0.0361). (**D**) The SD of the solidity (log rank test: *p* < 0.0001).

The results of the univariate cox regression for the categorical and dichotomized prognostic tests are provided in Table 4 and listed in Supplementary Table S6 for the numerical tests, showing that the hazard ratios are markedly higher for the morphometry than for the other prognostic tests. Notably, the conventional prognostic tests showed no discriminatory ability in the prognostication of cPC (Table 4 and Supplementary Table S10). For the anisokaryosis and shape irregularity estimates, the hazard ratios were highly variable between the individual pathologists (Supplementary Table S9). The Kaplan–Meier curves show that the prognostic thresholds can distinguish the patient outcome for the selected morphometric parameters determined by the algorithm (Figure 6) and manual method (Supplementary Figure S5).

## 4. Discussion

We were able to validate our hypotheses as follows and discuss them in detail below:

1. We have demonstrated the low inter-observer reproducibility of nuclear pleomorphism estimates among pathologists in cPC. Nuclear morphometry presents a reasonable approach to overcome this limitation.
2. The algorithm’s segmentation ability was good for nuclear size parameters and acceptable for shape parameters. Future studies may benefit from implementing filters and/or optimizing the algorithm to improve the model’s accuracy.
3. Regarding tumor-specific survival, the prognostic ability of the algorithmic morphometry was similar to manual morphometry for most parameters, with the exception of SD of solidity, and other established prognostic tests (histological grade, mitotic count, and clinical staging). The algorithm has the advantage of efficiency as a large number of nuclei can be measured within a few seconds and thus makes morphometry feasible for routine diagnostic service.

### 4.1. Inter-Observer Reproducibility

The anisokaryosis estimates by the pathologists had a similar prognostic value to the algorithmic and manual morphometry. The individual pathologist’s sensitivity and specificity values were similar to the respective points of the ROC curve for morphometry. However, the individual pathologists interpreted anisokaryosis differently, resulting in highly variable sensitivity and specificity values. The reasons for the variable classification of the anisokaryosis and nuclear shape between pathologists may include difficulties with clarity/variable interpretations of the methods including the number/percentage of nuclei that need to have two-fold size variation to warrant designation of high-score anisokaryosis. We have shown that different pathologists associate the same degree of SD of the nuclear area with different anisokaryosis scores and a previous study has shown similar results for canine mast cell tumors [40]. In addition, the pathologists have variable visual experience in nuclear size evaluation, which is particularly difficult in round-to-oval structures, i.e., nuclei, as the diameter is not proportional to the area. Improved methods for size and shape estimates, such as exemplary images in publications, reference sizes in digital images, etc., as previously used in lymphoma subtype classification [45] require further investigation in cPC. Updated definitions of the nuclear pleomorphism categories need to be explored given that estimates remain the only method available for light microscopy. In contrast, digital images/microscopy and nuclear morphometry hold high potential to standardize the assessment of nuclear characteristics and enhance the relevance of nuclear characteristics as a prognostic test, particularly when large numbers of nuclei are measured through automated methods. Studies have demonstrated that manual morphometry requires up to 15 min per image, which precludes its implementation in daily diagnostic practice [23,27]. Algorithmic approaches of morphometry performed by deep learning, as the current state of the art in nuclear segmentation, can overcome this limitation while remaining time-efficient and with high reproducibility.

However, future studies should evaluate the heterogeneity of nuclear characteristics throughout the tumor section and the effect on case interpretation when different ROIs are selected for algorithmic analysis. In our study, the evaluation of one or three tumor regions resulted in a similar ability to prognosticate patient outcome, suggesting a minor impact of tumor heterogeneity on reproducibility, but a more thorough analysis is warranted.

### 4.2. Test Accuracy of Algorithmic Morphometry

The ability of a segmentation model to accurately detect and identify objects of interest, in this case, nuclei, is crucial for its deployment in clinical use, particularly in terms of prognostic value. While statistical metrics indicated a good segmentation performance of the developed algorithm, the nuclear size measurements (based on the segmented nuclei) had a high similarity to the ground truth as determined by the RSME. However, the nuclear shape parameters (particularly solidity) were less accurate. A limitation of our study was the low number of test cases available for each model (*n* = 7), which was the motivation to conduct a cross validation with three separately trained segmentation models, providing a better impression of the generalization performance of our approach. A particular difficulty for cPC seems to be the close spatial connection of neoplastic nuclei and the overlap of nuclei in thick tissue sections, challenging algorithms to accurately separate individual nuclei. We investigated several filters (postprocessing of algorithmic segmentation masks) to reduce the impact of the insufficient separation of nuclei (undersegmentation). However, even with these approaches, we were unable to improve the overall performance (unpublished data). Filters to remove undersegmentation (i.e., several nuclei detected as one) may potentially be based on the abnormal large size or shape of the detected objects. Both of these features also represent the malignancy criteria of interest in the present study, thus explaining the difficulty in developing reasonable filters. The ability of our algorithm to distinguish neoplastic from non-neoplastic ones, such as fibrocytes and inflammatory cells, seemed to be appropriate; however, the analyzed images were selected to contain mostly tumor cells and a more detailed investigation is needed.

### 4.3. Inter-Algorithmic Reproducibility

When comparing the three algorithms based on the separately trained segmentation models, the inter-algorithmic consistency of the prognostic ability was good, with some variability in the AUC values for the outcome dataset. A direct comparison of the three models on the test subset of the ground truth dataset is not possible, as different test cases were used for each model. While the similarity on the outcome dataset confirms the algorithm’s training effectiveness, it should be noted that the ground truth dataset used for model development has some influence on the performance. Training a model is a statistical process (such as the random sampling of images for training), and thus, the variability between the models may be related to these random effects. Each model used different test cases, and variable test performance of the models may also be related to variable tissue quality between the different cases of the distinct subsets as proper fixation and section thickness may play a role.

### 4.4. Two-Dimensional vs. Stereological Approach

Nuclear morphometry can be performed by two methods: (1) three-dimensional (stereological) estimates of nuclear volume and (2) two-dimensional measurements of nuclear sections. As morphometry is influenced by the position and orientation of the nucleus, the stereological approach has been proposed as more representative [27,46]. It has been argued that the assumption of the correct position and orientation of the nucleus in two-dimensional measurements leads to an increased chance of measuring large nuclei more frequently [27]. However, the stereological approach has limitations, particularly the restriction to nuclear volume as the only assessable morphometric parameter. Thus, we have decided to use the two-dimensional approach. Due to the high number of nuclei available in each image, we consider the nuclei included in two-dimensional measurements sufficiently representative, allowing for the calculation of an informative probability density function. The advantage of two-dimensional measurements is the additional morphometric parameters that can be extracted from the segmentation maps, such as the mean, SD, and 90th percentile of the nuclear area. Parameters such as the 90th percentile and the percentage of large cells may be less prone to the variability in two-dimensional measurements depending on the orientation of the nucleus.

### 4.5. Prognostic Value

The morphometry of the size parameters showed good capability of discriminating cPC patients with favorable and unfavorable outcomes. The statistical results (such as the AUC and hazard ratios) showed a strong similarity between the algorithm and manual measurements, indicating that the algorithm is similar to manual measurements in predicting patient survival time. It should be noted that established prognostic indicators for cPC including the mitotic count [4,15], histological grade [7,16], and stage [7,10,14,16] had no prognostic value in our study population, supporting the prognostic importance of nuclear characteristics in this tumor type. In accordance with the findings of previous research conducted by Haghofer et al. [40], our study reaffirms the superior reproducibility and prognostic utility of automated nuclear morphometry over manual estimates by pathologists. Both studies demonstrate that algorithmic approaches offer enhanced reproducibility and time efficiency, when compared to manual morphometry, which is crucial for use in a routine diagnostic setting. In particular, Haghofer et al. reported an AUC of 0.943 for the SD of the nuclear area, assessed by automated morphometry, underscoring the predictive power of different area parameters, as we also found. Development and evaluation using similar algorithms for other aggressive tumor types should be investigated in the future.

The morphometric nuclear shape parameters mostly did not discriminate patient outcome. It should be noted that there is a large discrepancy between the AUC and hazard ratio values for the SD of the solidity, (i.e., shape irregularity) between the algorithm and manual morphometry, the reason for which was not apparent to us. While the SD of the solidity was the best prognostic test for algorithmic morphometry, it had no prognostic relevance when determined with manual morphometry. One potential explanation for this is that the SD of the solidity occurs locally in a specific tumor region and the manual measurement, evaluating only a restricted area of the sample (>100 nuclei), may not capture the SD of solidity representative for the tumor. However, discrepancies in the manual and algorithmic delineation of nuclei cannot be ruled out, particularly considering the high measurement error for this parameter (see above). Thus, the results of the SD of solidity should be interpreted with caution and further investigation of this parameter is needed.

The limitations of our study with regard to the outcome analysis are the small study population and the restriction of the outcome analysis to a follow-up period of 250 days. The small population size is due to the rarity of cPC, which significantly limits the number of available cases for a retrospective analysis. Our study includes all the available cases from two large veterinary pathology archives, representing the most comprehensive dataset we could assemble. Despite the limited sample size, our statistical analysis accounted for this constraint and yielded significant results. The 250-day follow-up period was chosen to account for a high lost-to-follow-up rate and to reduce competing risks in elderly dogs. Furthermore, the majority of patients received chemotherapy based on the results of staging and histological grading, which may have prolonged the survival time of patients with high-grade and advanced disease. We strongly encourage future studies to validate this newly developed diagnostic test in independent study populations with larger sample sizes and longer follow-up periods through multi-center collaborations. This will enable the validation and extension of our findings, which is needed before the test can be used in a diagnostic setting.

### 4.6. Diagnostic Applications and Considerations

Deep learning-based tools, like nuclear morphometry algorithms, have been increasingly investigated for numerous diagnostic/prognostic tasks in veterinary and human pathology in the detection [47,48], classification [24,49,50,51], and grading of tumors [52,53]. These studies have proposed different ways for algorithms to be implemented into the diagnostic workflow (fully automated and computer-assisted diagnosis). Considering the potential error of nuclear undersegmentation, resulting in excessive nuclear size measurements, we recommend verification of the segmentation mask by a trained pathologist when these algorithms are applied to diagnostic cases. In our study, we have used uncorrected algorithmic predictions and thus acknowledge potential errors particularly with regard to the overestimation of large nuclei. Different levels of human–machine interaction are imaginable to guarantee a correct prognostic interpretation of algorithmic morphometry, which should be evaluated in future studies.

## 5. Conclusions

Nuclear anisokaryosis and shape irregularity estimates by pathologists are hampered by marked inter-observer variability. Nuclear morphometry offers the potential to enhance the relevance of nuclear characteristics as a prognostic test for cPC by improving reproducibility. In our study, algorithmic and manual nuclear morphometry had a superior prognostic ability as compared to other histological tests. The developed algorithm (in contrast to manual methods) enables a time efficient implementation for routine tumor evaluation in laboratories with a digital workflow while maintaining at least a comparable performance. However, our study faced two primary limitations: a small study population and a short follow-up period. These new opportunities for the prognostication of the patient outcome in cPC, revealed by this study, warrant further validation in a larger and more diverse independent study population, as deep learning models rely heavily on data for training. Multi-center collaborations could help increase the sample size and diversity, thus providing a more robust validation of our findings. Furthermore, it is recommended that the algorithm be further refined, particularly with regard to the issue of the undersegmentation of nuclei, in order to enhance the reliability and utility of deep learning-based algorithms in veterinary pathology. This study establishes a foundation for future research in the use of deep learning-based techniques in veterinary pathology. However, addressing the identified limitations and ensuring the robustness of the used algorithm in various settings remains a critical consideration.

## Figures and Tables

**Table 1:** List of morphometric parameters used for algorithmic and manual morphometry.

Feature	Measurement	Parameters
Size	Area (in µm^2^)	Mean, median, standard deviation (SD), skewness, mean and median of the largest 10% of the nuclei, 90th percentile (90th P), percentage of large nuclei (>42.3 µm^2^ or >50.5 µm^2^)
Shape	Eccentricity	Mean, median, SD, skewness
	Solidity	Mean, median, SD, skewness

**Table 2:** The quality measures of the three segmentation models including the segmentation and object localization performance determined on 7 separate test images for each model.

Model	Binary Segmentation	Object Localization		
	Dice	F1	Precision	Recall
1	0.8073	0.8615	0.8877	0.8369
2	0.7761	0.8397	0.8726	0.8091
3	0.7801	0.8649	0.8470	0.8835

**Table 3:** The sensitivity, specificity, and precision regarding the tumor-related mortality at 250 days after surgery for the selected morphometric parameters measured by algorithmic (based on model 1) and manual morphometry in ROI 1. The analysis is based on 10 cases with tumor-specific mortality within the first 250 days after surgery and 25 cases that survived the follow-up period.

Morphometric Parameter	Method	Threshold Value	Sensitivity	Specificity	Precision
SD of area	Algorithmic	16 µm^2^	70.0%	68.0%	46.7%
	Manual	10 µm^2^	70.0%	64.0%	43.8%
Mean area of largest 10% of the nuclei	Algorithmic	70.5 µm^2^	70.0%	72.0%	50.0%
	Manual	53.03 µm^2^	70.0%	64.0%	43.8%
		50 µm^2^	90.0%	64.0%	50.0%
Percentage of nuclei above 42.3 µm^2^	Algorithmic	19%	70.0%	68.0%	46.7%
	Manual	26%	70.0%	76.0%	53.8%
SD of solidity	Algorithmic	0.045	70.0%	92.0%	77.8%
	Manual	0.0204	70.0%	48.0%	35.0%

**Table 4:** Hazard ratios (HR) with 95% confidence intervals (95%CI; univariate cox regression) for categorical and dichotomized prognostic tests. Tumor-specific survival was tested with a follow-up period of up to 250 days after surgery.

	Prognostic Test	Algorithm		Pathologists	
		Threshold	HR (95%CI)	Threshold/Categories	HR (95%CI)
Morphometry	SD of area	16 µm^2^	3.38 (0.87–13.1)	10 µm^2^	3.32 (0.85–12.9)
	Mean area of largest 10% of nuclei	70.5 µm^2^	3.85 (0.99–15.0)	50 µm^2^	9.83 (1.24–78.1)
	% nuclei above 42.3 µm^2^	19%	3.32 (0.85–12.9)	26%	4.34 (1.12–16.9)
	SD of solidity	0.045	9.88 (2.52–38.7)	0.0204	1.94 (0.50–7.52)
Other	Grade	NA	NA	1 vs. 2, 3	1.01 (0.12–7.98)
		NA	NA	1, 2 vs. 3	1.75 (0.36–8.36)
	MC, pathologist 1	NA	NA	1, 2 vs. 3, 4	0.79 (0.20–3.08)
	MC, pathologist 2	NA	NA	1, 2 vs. 3, 4	0.41 (0.10–1.61)
	Stage	NA	NA	1 vs. 2, 3, 4	1.42 (0.36–5.49)

## Data Availability

The original contributions presented in this study are included in the article/Supplementary Material; further inquiries can be directed to the corresponding author.

## Supplementary Materials

The following supporting information can be downloaded at: https://www.mdpi.com/article/10.3390/vetsci11060278/s1, **Figure S1.** Nuclear segmentation of model 1 in an exemplary case. By applying the neural network on the original HE image (on the left), the nuclei were segmented (binary mask on the right), which resulted in a Dice score of 0.838 as compared to the ground truth (binary mask in the middle).; **Figure S2.** The comparison between the measurements of the algorithmic nuclear morphometry (models 1–3) with the ground truth measurements for the 21 test cases of the ground truth dataset. Scatter plots for the (A) standard deviation (SD) of the nuclear area, (B) mean area of the largest 10% of the nuclei, (C) mean nuclear area, (D) 90th percentile of nuclear area, (E) percentage of nuclei with an area >42.3 µm², and (D) SD of the solidity.; **Figure S3.** Area under the ROC curve (AUC) values (tumor-related mortality) comparing results for algorithmic morphometry (algorithm 1–3) of ROI 1 with morphometry (algorithms 1–3) of ROIs 1–3 for selected parameters. SD, standard deviation.; **Figure S4.** The scatter plots of the morphometric parameters comparing the cases with tumor-related mortality (TRM) at 250 days after surgery with the cases that survived this follow-up period. The upper row (A–D) depicts the results of the algorithmic morphometry (based on model 1) and the lower row the results of the manual morphometry (E–H) determined in region of interest (ROI) 1. (A) and (E) are the standard deviation (SD) of the nuclear area, (B) and (F) the mean of the largest 10% of the nuclei, (C) and (G) the percentage of the nuclei with an area >42.2 µm², and (D) and (H) the SD of the solidity. The dotted line represents the selected prognostic threshold values. The analysis is based on 10 cases with tumor-specific mortality within the first 250 days after surgery and 25 cases that survived this follow-up period.; **Figure S5.** The Kaplan–Meier curves for the tumor-specific survival time (until 250 days after surgery) for the different nuclear size and shape parameters of the manual morphometry. The analysis is based on 46 cases with the censoring of the cases that were lost to follow-up or died due to tumor-unrelated causes (*n* = 11). A total of 10 cases died of tumor-related causes and 25 dogs survived the follow-up period. A) The standard deviation (SD) of the nuclear area (log rank test: *p* = 0.0658). B) The percentage of nuclei > 42.3 µm² (log rank test: *p* = 0.0211). C) The mean area of the largest 10% of the nuclei (log rank test: *p* = 0.0080). D) The SD of the solidity (log rank test: *p* = 0.3280). **Table S1.** The root mean square error (RMSE) of the measured morphometric nuclear size parameters separately for the three deep learning-based algorithms (Algorithm) with 7 test cases of the ground truth dataset used for each algorithm.; **Table S2.** The root mean square error (RMSE) of the measured morphometric nuclear shape parameters separately for the three deep learning-based algorithms with 7 test cases of the ground truth dataset used for each algorithm.; **Table S3.** The area under the ROC curve (AUC) regarding the tumor-related mortality at 250 days after surgery for the nuclear morphometry based on the three deep learning-based algorithms and manual measurements determined in the region of interest (ROI) 1. The analysis is based on 10 cases with tumor-specific mortality within the first 250 days after surgery and 25 cases that survived 250 days after surgery.; **Table S4.** The area under the ROC curve (AUC) regarding the all-cause mortality at 250 days after surgery for the nuclear morphometry based on the three deep learning-based algorithms and manual measurements determined in the region of interest (ROI) 1. The analysis is based on 13 cases with death (any cause) within the first 250 days after surgery and 25 cases that survived 250 days after surgery.; **Table S5.** The area under the ROC curve (AUC) regarding the tumor-related mortality at 250 days after surgery for the nuclear morphometry based on the three deep learning-based algorithms determined in the regions of interest (ROIs) 1–3. The analysis is based on 10 cases with tumor-specific mortality within the first 250 days after surgery and 25 cases that survived 250 days after surgery.; **Table S6.** The hazard ratios (determined from z-standardized * numerical values) for the morphometric parameter measurements performed by the algorithmic model 1 and the manual measurement in ROI 1. The analysis is based on 46 cases with the censoring of cases that were lost to follow-up or died due to tumor-unrelated causes (*n* = 11). A total of 10 cases died of tumor-related causes and 25 dogs survived the follow-up period.; **Table S7.** The sensitivity, specificity, and precision regarding the tumor-related mortality at 250 days after surgery for the three-tier estimates of anisokaryosis by 11 pathologists. The analysis is based on 10 cases with tumor-specific mortality within the first 250 days after surgery and 25 cases that survived this follow-up period.; **Table S8.** The sensitivity, specificity, and precision regarding the tumor-related mortality at 250 days after surgery for the two-tier estimates of the nuclear shape irregularity by the pathologists. The analysis is based on 10 cases with tumor-specific mortality within the first 250 days after surgery and 25 cases that survived 250 days after surgery.; **Table S9.** Hazard ratios for estimates of anisokaryosis (three-tier) and nuclear shape irregularity (two-tier) regarding tumor-specific survival with a follow-up period of up to 250 days after surgery.; **Table S10.** The sensitivity, specificity, and precision regarding the tumor-related mortality at 250 days after surgery for the histologic grade, mitotic count, and clinical staging. The analysis is based on 10 cases with tumor-specific mortality within the first 250 days after surgery and 25 cases that survived 250 days after surgery.; **Table S11.** Histological grading characteristics and corresponding definitions, descriptions, and scores as suggested by McNiel et al. (J Am Vet Med Assoc. 1997, 1;211(11):1422-7).
