# Supplementary material for: Automated Nuclear Morphometry: A Deep Learning Approach for Prognostication in Canine Pulmonary Carcinoma to Enhance Reproducibility
Source: Vet Sci. 2024 Jun 17;11(6):278. doi: 10.3390/vetsci11060278 (PMC11209399; doi:10.3390/vetsci11060278)
Supplement: Supplementary file 1 [file vetsci-11-00278-s001.zip › vetsci-2994029-supplementary.pdf]

Supplemental material

Glahn et al. Nuclear Morphometry using a Deep Learning-based Algorithms has Prognostic Relevance for Canine Pulmonary Carcinoma

Method accuracy

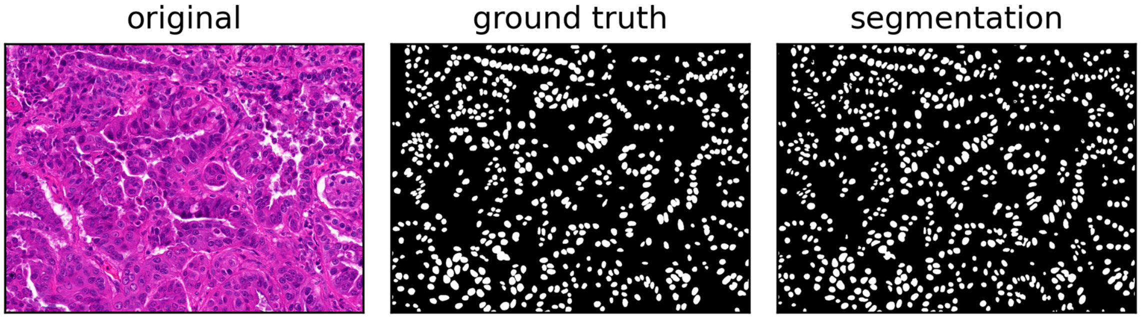

Supplemental Figure S1. Nuclear segmentation of model 1 in an exemplary case. By applying the neural network on the original HE image (on the left), the nuclei were segmented (binary mask on the right), which resulted in a Dice score of 0.838 as compared to the ground truth (binary mask in the middle).

Supplemental Table S1. The root mean square error (RMSE) of the measured morphometric **nuclear size parameters** separately for the three deep learning-based algorithms (Algorithm) with 7 test cases of the ground truth dataset used for each algorithm.

Morphometric parameter	Algorithm	RMSE	Value range of the ground truth measurements	RMSE-to-range ratio (coefficient of variation)
SD of area	1	3.276 μm²	6.380 - 13.410 μm²	46.6%
	2	1.421 μm²	5.007 - 18.665 μm²	10.4%
	3	0.582 μm²	5.766 - 12.227 μm²	7.8%
Mean area	1	2.327 μm²	21.292 - 39.219 μm²	13.0%
	2	3.317 μm²	15.839 - 56.207 μm²	8.2%
	3	3.679 μm²	22.764 - 34.437 μm²	31.5%
Median area	1	2.384 μm²	19.847 - 38.025 μm²	13.1%
	2	4.981 μm²	15.024 - 55.924 μm²	12.2%
	3	3.497 μm²	22.135 - 32.769 μm²	32.9%
Skewness of area	1	1.391	0.615 - 2.669	67.7%
	2	0.413	0.110 - 1.442	31.0%
	3	0.326	0.498 - 1.130	51.7%
Mean of the largest 10% of the nuclei	1	7.742 μm²	37.718 - 65.082 μm²	28.3%
	2	2.528 μm²	26.162 - 89.727 μm²	4.0%
	3	3.720 μm²	34.331 - 63.097 μm²	12.9%
Median of the largest 10% of the nuclei	1	5.828 μm²	35.119 - 60.159 μm²	23.3%
	2	3.488 μm²	25.164 - 89.250 μm²	5.4%
	3	4.038 μm²	33.542 - 60.036 μm²	15.2%
90th percentile	1	2.878 μm²	30.061 - 54.817 μm²	11.6%
	2	3.444 μm²	22.877 - 80.903 μm²	5.9%
	3	4.246 μm²	30.333 - 51.949 μm²	19.6%
Percentage of nuclei >42.3 μm²	1	1.920 %	0.797 - 38.671 %	5.1%
	2	7.536 %	0 - 75.245 %	10.0%
	3	3.527 %	0.135 - 22.969 %	15.4%
Percentage of nuclei >50.5 μm²	1	1.892 %	0.478 - 15.843 %	12.3%
	2	8.183 %	0 - 62.637 %	13.1%
	3	1.934 %	0 - 10.644 %	18.2%

SD, standard deviation.

Supplemental Table S2. The root mean square error (RMSE) of the measured morphometric **nuclear shape parameters** separately for the three deep learning-based algorithms with 7 test cases of the ground truth dataset used for each algorithm.

Morphometric parameter	Algorithm	RMSE	Value range of the ground truth measurements	RMSE-to-range ratio (coefficient of variation)
SD of eccentricity	1	0.011	0.141 - 0.156	75.6%
	2	0.008	0.139 - 0.154	51.0%
	3	0.004	0.139 - 0.150	35.5%
SD of solidity	1	0.014	0.018 - 0.035	80.5%
	2	0.009	0.016 - 0.033	51.0%
	3	0.011	0.017 - 0.024	155.2%
Mean eccentricity	1	0.151	0.583 - 0.715	11.4%
	2	0.018	0.065 - 0.733	21.2%
	3	0.015	0.636 - 0.684	30.4%
Mean solidity	1	0.005	0.948 - 0.961	41.2%
	2	0.007	0.934 - 0.963	22.3%
	3	0.003	0.951 - 0.958	42.3%
Median eccentricity	1	0.013	0.586 - 0.742	8.1%
	2	0.019	0.668 - 0.764	20.4%
	3	0.020	0.656 - 0.701	43.6%
Median solidity	1	0.002	0.955 - 0.964	29.4%
	2	0.006	0.939 - 0.968	19.1%
	3	0.003	0.954 - 0.961	47.7%
Skewness of eccentricity	1	0.112	-1.041 - -0.078	11.6%
	2	0.104	-0.967 - -0.549	24.9%
	3	0.155	-0.728 - -0.456	57.0%
Skewness of solidity	1	2.014	-6.378 - -4.116	89.0%
	2	3.415	-12.252 - -2.099	33.6%
	3	4.431	-13.657 - -4.058	46.2%

SD, standard deviation.

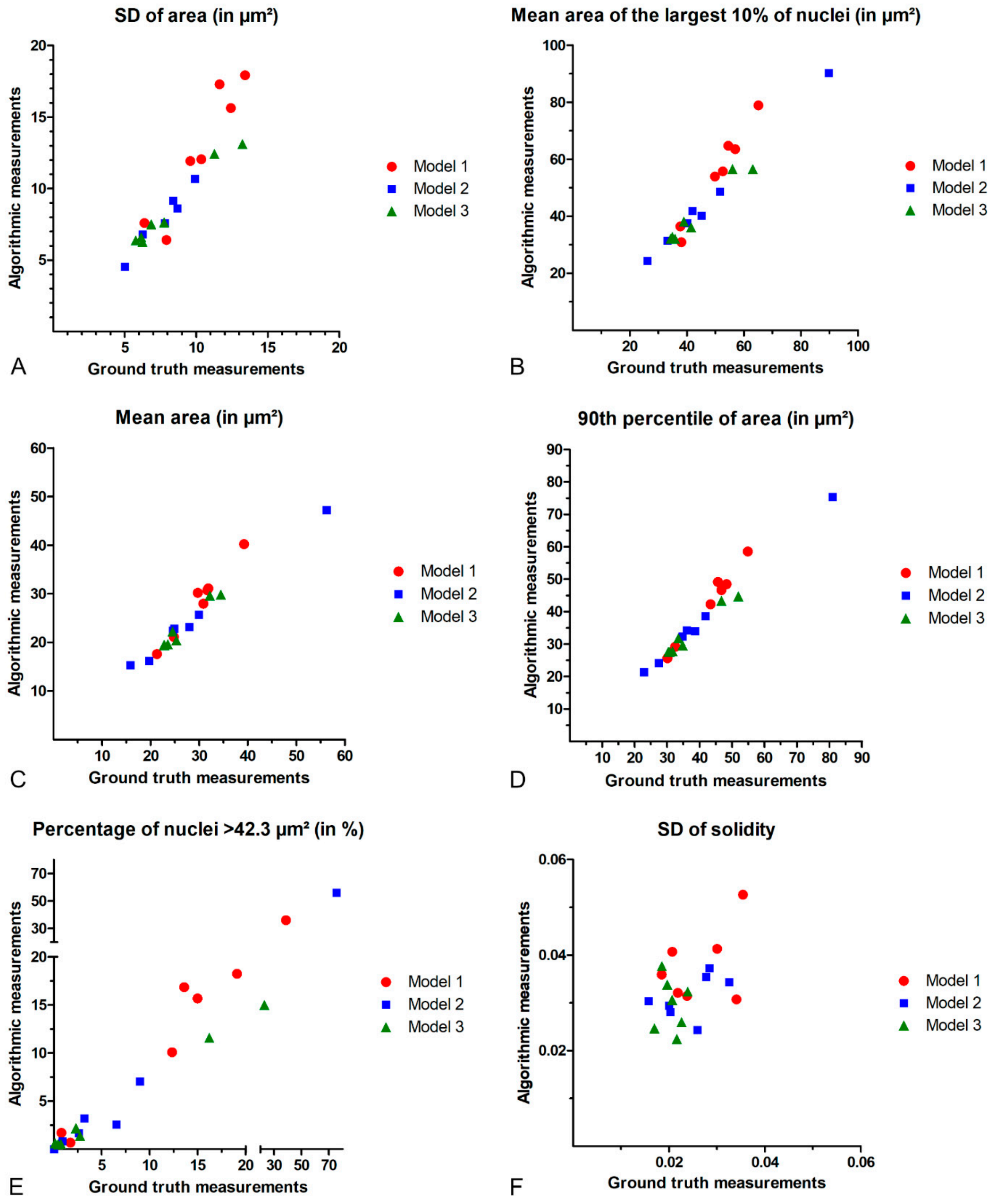

Supplemental Figure S2. The comparison between the measurements of the algorithmic nuclear morphometry (models 1-3) with the ground truth measurements for the 21 test cases of the ground truth dataset. Scatter plots for the (A) standard deviation (SD) of the nuclear area, (B) mean area of the largest 10% of the nuclei, (C) mean nuclear area, (D) 90th percentile of nuclear area, (E) percentage of nuclei with an area >42.3 µm², and (D) SD of the solidity.

Prognostic value of nuclear morphometry (algorithmic and manual)

Demographic information of the cases from the outcome dataset

Among the 46 dogs included in the outcome dataset, there were 20 neutered females, 4 intact females, 19 neutered males, and 4 intact males. The breeds represented included Mixed (N = 10), Maltese (N = 4), Boxer (N = 3), Jack Russell Terrier (N = 3), and other breeds (N = 26). The ages ranged from 6 to 14 years, with a median age of 10.9 years and an interquartile range of 10 to 12 years. Most of the dogs (N = 36, 78%) were older than 10 years. In terms of treatment, 25 dogs underwent surgery alone, while 19 dogs received surgery and additional chemotherapy. Besides curative-intent surgery in all the cases, chemotherapy was applied in 19 cases using carboplatin (N = 13), vinorelbine (N = 9), toceranib (N = 7), and metronomic (N = 1). Chemotherapy was more common in cases with a higher grade, i.e., 1/6 (17%), 14/32 (44%), and 4/8 (50%) of grade 1, 2, and 3, respectively.

Area under the curve

Supplemental Table S3. The area under the ROC curve (AUC) regarding the **tumor-related mortality at 250 days** after surgery for the nuclear morphometry based on the three deep learning-based algorithms and manual measurements determined in the region of interest **(ROI) 1**. The analysis is based on 10 cases with tumor-specific mortality within the first 250 days after surgery and 25 cases that survived 250 days after surgery.

Morphometric Parameter	AUC (95% confidence interval)			
	**Algorithm 1**	**Algorithm 2**	**Algorithm 3**	**Manual**
SD of area	0.800 (0.645 -0.955)	0.784 (0.624 - 0.944)	0.772 (0.610 - 0.934)	0.764 (0.593 - 0.935)
Mean area	0.772 (0.610 -0.934)	0.784 (0.626 - 0.942)	0.776 (0.615 - 0.937)	0.812 (0.668 - 0.956)
Median area	0.764 (0.598 -0.930)	0.760 (0.594 - 0.924)	0.770 (0.606 - 0.934)	0.816 (0.673 - 0.959)
Skewness of area	0.724 (0.528 -0.920)	0.704 (0.505 - 0.903)	0.628 (0.413 - 0.843)	0.408 (0.189 - 0.627)
Mean of the largest 10% of the nuclei	0.796 (0.642 -0.946)	0.792 (0.637 - 0.947)	0.776 (0.615 - 0.937)	0.784 (0.627 - 0.941)
Median of the largest 10% of the nuclei	0.790 (0.634 -0.950)	0.784 (0.627 - 0.941)	0.776 (0.615 - 0.937)	0.794 (0.640 - 0.948)
90th percentile of area	0.768 (0.604 -0.932)	0.772 (0.611 - 0.933)	0.772 (0.611 - 0.933)	0.808 (0.660 - 0.956)
Percentage of nuclei >42.3 μm^2	0.772 (0.609 -0.935)	0.772 (0.613 - 0.931)	0.780 (0.620 - 0.940)	0.808 (0.661 - 0.955)
Percentage of nuclei >50.5 μm^2	0.764 (0.600 -0.928)	0.756 (0.590 -0-922)	0.776 (0.615 - 0.937)	0.796 (0.645 - 0.945)
SD of eccentricity	0.660 (0.457 -0.863)	0.648 (0.435 - 0.861)	0.700 (0.525 - 0.875)	0.588 (0.385 - 0.791)
SD of solidity	0.824 (0.666 -0.982)	0.668 (0.473 - 0.863)	0.800 (0.648 - 0.952)	0.556 (0.360 - 0.752)
Mean eccentricity	0.392 (0.184 -0.600)	0.372 (0.163 - 0.581)	0.376 (0.177 - 0.575)	0.500 (0.288 - 0.712)
1—Mean solidity	0.624 (0.415 -0.833)	0.508 (0.281 - 0.735)	0.568 (0.354 - 0.782)	0.276 (0.085 - 0.467)
Skewness of eccentricity	0.692 (0.485 -0.899)	0.652 (0.466 - 0.838)	0.712 (0.531 - 0.893)	0.472 (0.238 - 0.706)
Skewness of solidity	0.692 (0.512 -0.872)	0.780 (0.612 - 0.948)	0.788 (0.633 - 0.943)	0.348 (0.140 - 0.556)

SD, standard deviation.

Supplemental Table S4. The area under the ROC curve (AUC) regarding the **all-cause mortality at 250 days** after surgery for the nuclear morphometry based on the three deep learning-based algorithms and manual measurements determined in the region of interest **(ROI) 1**. The analysis is based on 13 cases with death (any cause) within the first 250 days after surgery and 25 cases that survived 250 days after surgery.

**Morphometric Parameter**	**AUC (95% confidence interval)**			
	**Algorithm 1**	**Algorithm 2**	**Algorithm 3**	**Manual**
SD of area	0.685 (0.508 -0.861)	0.658 (0.473 - 0.842)	0.664 (0.484 - 0.843)	0.679 (0.501 -0.857)
Mean area	0.670 (0.491 -0.848)	0.673 (0.495 - 0.850)	0.667 (0.490 - 0.844)	0.711 (0.544 -0.879)
Median area	0.670 (0.493 -0.846)	0.667 (0.492 - 0.842)	0.665 (0.489 - 0.842)	0.699 (0.528 -0.870)
Skewness of area	0.646 (0.457 -0.834)	0.586 (0.388 - 0.785)	0.536 (0.337 - 0.734)	0.470 (0.269 -0.672)
Mean of the largest 10% of the nuclei	0.682 (0.507 -0.856)	0.670 (0.491 - 0.849)	0.667 (0.488 - 0.846)	0.688 (0.512 -0.863)
Median of the largest 10% of the nuclei	0.685 (0.511 -0.858)	0.673 (0.495 - 0.850)	0.661 (0.478 - 0.843)	0.689 (0.513 -0.865)
90th percentile of area	0.673 (0.496 -0.849)	0.667 (0.487 - 0.846)	0.679 (0.504 - 0.853)	0.699 (0.529 -0.870)
Percentage of nuclei >42.3 μm^2	0.673 (0.494 -0.851)	0.667 (0.486 - 0.847)	0.670 (0.489 - 0.850)	0.676 (0.494 -0.857)
Percentage of nuclei >50.5 μm^2	0.676 (0.501 -0.850)	0.649 (0.465 - 0.833)	0.679 (0.502 - 0.855)	0.720 (0.559 -0.882)
SD of eccentricity	0.616 (0.425 -0.807)	0.634 (0.445 - 0.823)	0.696 (0.527 - 0.866)	0.542 (0.355 -0.729)
SD of solidity	0.688 (0.503 -0.872)	0.533 (0.337 - 0.729)	0.667 (0.485 - 0.848)	0.497 (0.311 -0.683)
Mean eccentricity	0.396 (0.201 -0.591)	0.375 (0.181 - 0.569)	0.366 (0.178 - 0.554)	0.491 (0.287 -0.695)
1—Mean solidity	0.452 (0.256 -0.649)	0.446 (0.246 - 0.647)	0.506 (0.311 - 0.701)	0.262 (0.095 -0.429)
Median eccentricity	0.393 (0.198 -0.588)	0.366 (0.177 - 0.555)	0.372 (0.183 - 0.561)	0.470 (0.260 -0.681)
1—Median solidity	0.699 (0.507 -0.892)	0.271 (0.082 - 0.460)	0.330 (0.134 - 0.526)	0.286 (0.117 -0454)
Skewness of eccentricity	0.664 (0.473 -0.854)	0.667 (0.485 - 0.849)	0.702 (0.527 - 0.878)	0.518 (0.311 -0.725)
Skewness of solidity	0.577 (0.391 -0.764)	0.661 (0.477 - 0.844)	0.646 (0.458 - 0.833)	0.327 (0.146 -0.508)

SD, standard deviation.

Supplemental Table S5. The area under the ROC curve (AUC) regarding the **tumor-related mortality at 250 days** after surgery for the nuclear morphometry based on the three deep learning-based algorithms determined in the regions of interest **(ROIs) 1-3**. The analysis is based on 10 cases with tumor-specific mortality within the first 250 days after surgery and 25 cases that survived 250 days after surgery.

Morphometric Parameter	AUC (95% confidence interval)		
	Algorithm 1	Algorithm 2	Algorithm 3
SD of area	0.828 (0.675 -0.981)	0.788 (0.626 -0.950)	0.772 (0.610 -0.934)
Mean area	0.756 (0.594 -0.918)	0.752 (0.590 -0.914)	0.760 (0.600 -0.920)
Median area	0.740 (0.574 -0.906)	0.740 (0.575 -0.905)	0.740 (0.575 -0.905)
Skewness of area	0.784 (0.615 -0.953)	0.692 (0.503 -0.881)	0.624 (0.419 -0.829)
Mean of the largest 10% of the nuclei	0.808 (0.656 -0.960)	0.788 (0.635 -0.941)	0.780 (0.624 -0.936)
Median of the largest 10% of the nuclei	0.784 (0.625 -0.943)	0.784 (0.629 -0.939)	0.774 (0.617 -0.931)
90th percentile of area	0.764 (0.599 -0.929)	0.744 (0.579 -0.909)	0.752 (0.588 -0.916)
Percentage of nuclei >42.3 μm^2	0.716 (0.542 -0.890)	0.728 (0.559 -0.897)	0.728 (0.561 -0.895)
Percentage of nuclei >50.5 μm^2	0.732 (0.560 -0.904)	0.732 (0.563 -0.901)	0.748 (0.584 -0.912)
SD of eccentricity	0.588 (0.371 -0.805)	0.664 (0.473 -0.855)	0.612 (0.390 -0.834)
SD of solidity	0.816 (0.673 -0.959)	0.832 (0.680 -0.984)	0.860 (0.732 -0.988)
Mean eccentricity	0.532 (0.300 -0.764)	0.516 (0.291 -0.741)	0.516 (0.289 -0.743)
1—Mean solidity	0.704 (0.520 -0.888)	0.664 (0.474 -0.854)	0.656 (0.440 -0.872)
Median eccentricity	0.516 (0.286 -0.746)	0.520 (0.295 -0.745)	0.532 (0.301 -0.763)
1—Median solidity	0.352 (0.131 -0-573)	0.340 (0.118 -0.562)	0.352 (0.133 -0.571)
Skewness of eccentricity	0.516 (0.290 -0.742)	0.556 (0.344 -0.768)	0.540 (0.306 -0.774)
Skewness of solidity	0.808 (0.658 -0.958)	0.640 (0.433 -0.847)	0.836 (0.678 -0.994)

SD, standard deviation.

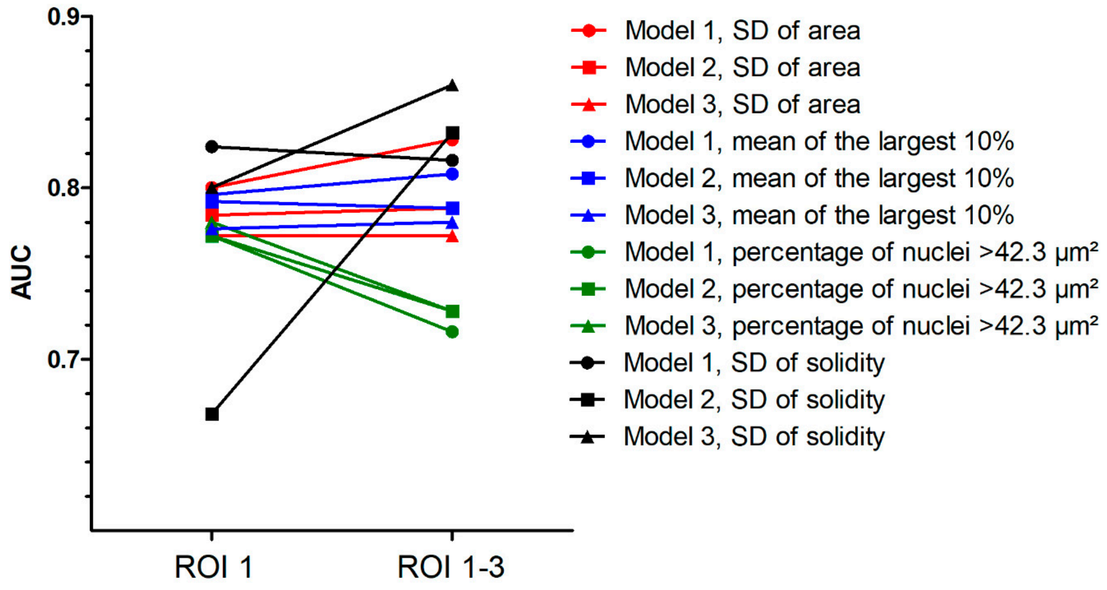

Supplemental Figure S3. Area under the ROC curve (AUC) values (tumor-related mortality) comparing results for algorithmic morphometry (algorithm 1-3) of ROI 1 with morphometry (algorithms 1-3) of ROIs 1-3 for selected parameters.
SD, standard deviation.

Scatter plots

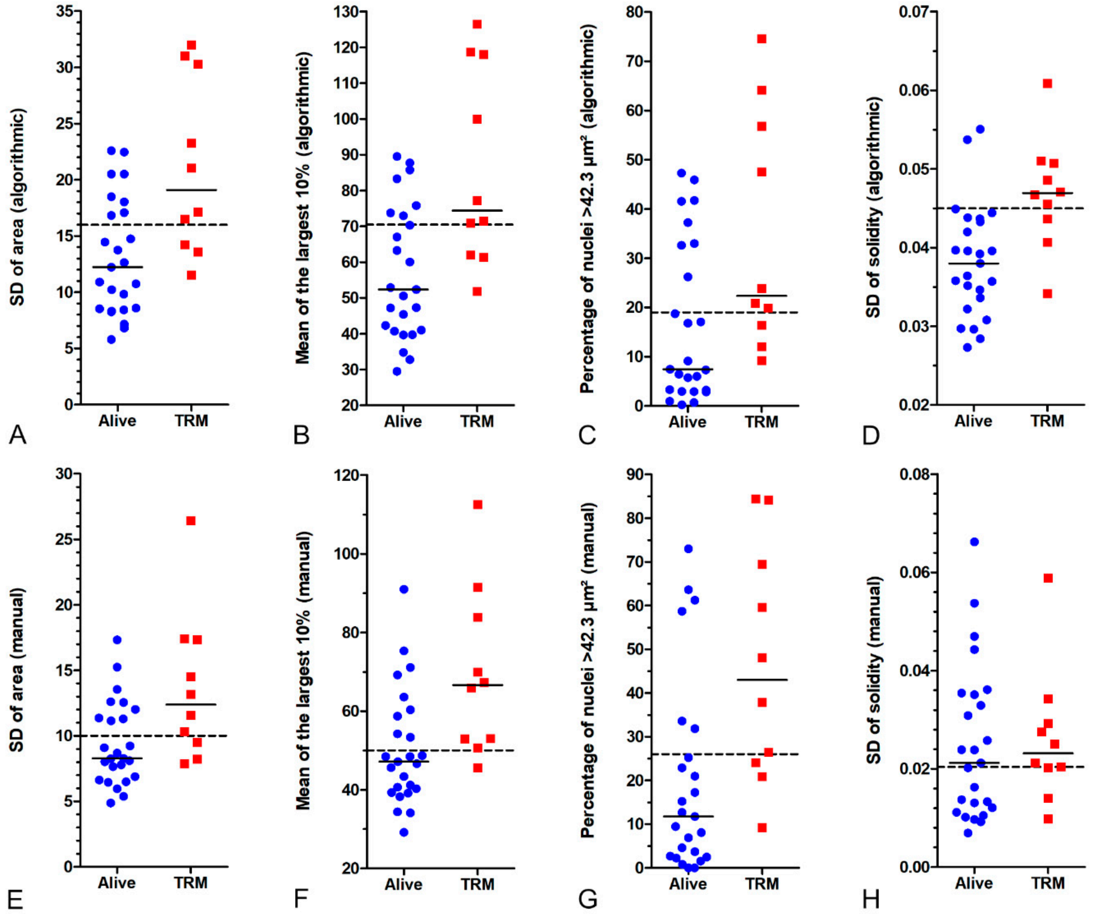

Supplemental Figure S4. The scatter plots of the morphometric parameters comparing the cases with tumor-related mortality (TRM) at 250 days after surgery with the cases that survived this follow-up period. The upper row (A – D) depicts the results of the algorithmic morphometry (based on model 1) and the lower row the results of the manual morphometry (E – H) determined in region of interest (ROI) 1. (A) and (E) are the standard deviation (SD) of the nuclear area, (B) and (F) the mean of the largest 10% of the nuclei, (C) and (G) the percentage of the nuclei with an area >42.2 µm², and (D) and (H) the SD of the solidity. The dotted line represents the selected prognostic threshold values. The analysis is based on 10 cases with tumor-specific mortality within the first 250 days after surgery and 25 cases that survived this follow-up period.

Hazard ratio and Kaplan–Meier curves

Supplemental Table S6. The hazard ratios (determined from z-standardized * numerical values) for the morphometric parameter measurements performed by the algorithmic model 1 and the manual measurement in ROI 1. The analysis is based on 46 cases with the censoring of cases that were lost to follow-up or died due to tumor-unrelated causes (N = 11). A total of 10 cases died of tumor-related causes and 25 dogs survived the follow-up period.

Morphometric parameter	Method	Hazard ratio (95% confidence interval, p-value)
SD of area	Algorithmic	2.161 (1.244 - 3.755, p = 0.006)
	Manual	1.625 (1.071 - 2.465, p = 0.023)
Mean area of largest 10% of nuclei	Algorithmic	2.165 (1.266 - 3.701, p = 0.005)
	Manual	1.770 (1.114 - 2.812, p = 0.016)
Percentage of nuclei >42.3 μm²	Algorithmic	1.730 (1.046 - 2.863, p = 0.033)
	Manual	2.004 (1.159 - 3.465, p = 0.013)
SD of solidity (values multiplied by 100)	Algorithmic	2.110 (1.193 - 3.734, p = 0.010)
	Manual	1.094 (0.758 - 1.936, p = 0.758)

* The z-standardization was performed in order to allow for comparability between the morphometric parameters using the following formula for the individual values determined for each case: z-score = (value - mean) / standard deviation.

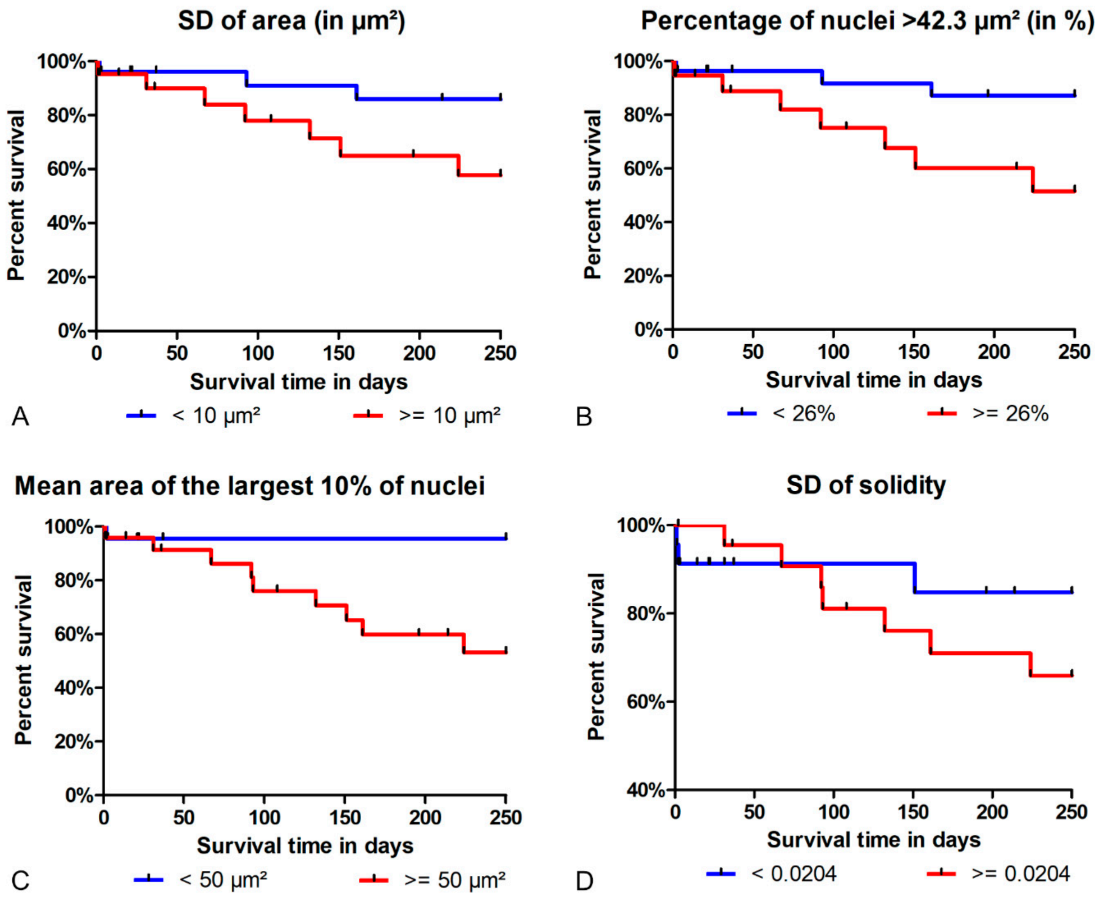

Supplemental Figure S5. The Kaplan–Meier curves for the tumor-specific survival time (until 250 days after surgery) for the different nuclear size and shape parameters of the manual morphometry. The analysis is based on 46 cases with the censoring of the cases that were lost to follow-up or died due to tumor-unrelated causes (N = 11). A total of 10 cases died of tumor-related causes and 25 dogs survived the follow-up period. A) The standard deviation (SD) of the nuclear area (log rank test: $p = 0.0658$). B) The percentage of nuclei > 42.3 µm² (log rank test: $p = 0.0211$). C) The mean area of the largest 10% of the nuclei (log rank test: $p = 0.0080$). D) The SD of the solidity (log rank test: $p = 0.3280$).

Prognostic value of estimates of anisokaryosis and nuclear irregularity

Supplemental Table S7. The sensitivity, specificity, and precision regarding the tumor-related mortality at 250 days after surgery for the three-tier estimates of **anisokaryosis** by 11 pathologists. The analysis is based on 10 cases with tumor-specific mortality within the first 250 days after surgery and 25 cases that survived this follow-up period.

**Pathologist**	**Anisokaryosis 1+2 vs. 3**			**Anisokaryosis 1 vs. 2+3**		
	**Sensitivity**	**Specificity**	**Precision**	**Sensitivity**	**Specificity**	**Precision**
1	0%	100%	NA	60.0%	84.0%	60.0%
2	20.0%	92.0%	50.0%	80.0%	48.0%	38.1%
3	60.0%	84.0%	60.0%	90.0%	36.0%	36.0%
4	50.0%	80.0%	50.0%	100%	8.0%	30.3%
5	20.0%	96.0%	66.7%	100%	36%	38.5%
6	20.0%	92.0%	50.0%	100%	32.0%	37.0%
7	10.0%	92.0%	33.3%	80.0%	64.0%	47.1%
8	90.0%	56.0%	45.0%	100%	0%	28.6%
9	40.0%	72.0%	36.4%	90.0%	48.0%	40.9%
10	20.0%	92.0%	50.0%	80.0%	64.0%	47.1%
11	20.0%	92.0%	50.0%	90.0%	28.0%	33.3%

NA, not available due to division by 0.

Supplemental Table S8. The sensitivity, specificity, and precision regarding the tumor-related mortality at 250 days after surgery for the two-tier estimates of the **nuclear shape irregularity** by the pathologists. The analysis is based on 10 cases with tumor-specific mortality within the first 250 days after surgery and 25 cases that survived 250 days after surgery.

Pathologist	Nuclear shape irregularity (absent vs. present)		
	Sensitivity	Specificity	Precision
1	10.0%	80.0%	16.7%
2	10.0%	40.0%	6.3%
3	70.0%	68.0%	46.7%
4	70.0%	16.0%	25.0%
5	60.0%	52.0%	33.3%
6	60.0%	48.0%	31.6%
7	70.0%	64.0%	43.8%
8	40.0%	40.0%	21.1%
9	20.0%	76.0%	25.0%
10	60.0%	76.0%	50.0%
11	20.0%	56.0%	15.4%

Supplemental Table S9. Hazard ratios for estimates of anisokaryosis (three-tier) and nuclear shape irregularity (two-tier) regarding tumor-specific survival with a follow-up period of up to 250 days after surgery.

Pathologist	Hazard ratio (95% confidence interval)		
	Anisokaryosis 1+2 vs. 3	Anisokaryosis 1 vs. 2+3	Nuclear shape irregularity
1	NA	5.38 (1.49 - 19.4, p = 0.010)	0.55 (0.06 - 4.33, p = 0.568)
2	2.11 (0.45 - 9.97, p = 0.344)	3.29 (0.69 - 15.5, p = 0.133)	0.11 (0.01 - 0.87, p = 0.037
3	1.67 (1.09 - 2.55, p = 0.018)	4.22 (0.53 - 33.4, p = 0.172)	3.53 (0.90 - 13.7, p = 0.068)
4	2.75 (0.79 - 9.58, p = 111)	22.1 (not converted, p = 0.597)	0.63 (0.16 - 2.45, p = 0.508)
5	4.01 (0.82 - 19.5, p = 0.084)	34.8 (not converted, p = 0.228)	1.47 (0.41 - 5.23, p = 0.552)
6	1.85 (0.39 - 8.74, p = 0.436)	32.3 (not converted, p = 0.259)	1.25 (0.35 - 4.41, p = 0.734)
7	0.86 (0.10 - 6.78, p = 0.885)	5.12 (1.08 - 24.2, p = 0.039)	3.29 (0.84 - 12.8, p = 0.086)
8	8.48 (1.071 - 67.1, p = 0.043)	20.7 (not converted, p = 0.888)	0.58 (0.16 - 2.05, p = 0.397)
9	1.36 (0.38 - 4.82, p = 0.636)	6.11 (0.77 - 48.4, p = 0.087)	0.94 (0.19 - 4.4, p = 0.933)
10	2.24 (0.47 - 10.5, p = 0.309)	4.75 (1.00 - 22.5, p = 0.050)	3.80 (1.06 - 13.6, p = 0.039)
11	1.74 (0.36 - 8.22, p = 0.485)	2.87 (0.36 - 22.7, p = 0.317)	0.43 (0.09 - 2.04, p = 0.290)

NA, not available due to the lack of cases classified in both categories.

Other prognostic tests

Supplemental Table S10. The sensitivity, specificity, and precision regarding the tumor-related mortality at 250 days after surgery for the histologic grade, mitotic count, and clinical staging. The analysis is based on 10 cases with tumor-specific mortality within the first 250 days after surgery and 25 cases that survived 250 days after surgery.

Prognostic test	Categories	Sensitivity	Specificity	Precision
Grade	1+2 vs. 3	20.0%	92.0%	50.0%
	1 vs. 2+3	90.0%	8.0%	28.1%
Mitotic count	1+2 vs. 3+4 (pathologist 1)	30.0%	64.0%	25.0%
	1+2 vs. 3+4 (pathologist 2)	30.0%	48.0%	18.8%
Stage	1 vs. 2+3+4	30.0%	76.0%	33.3%

Supplemental Table S11. Histological grading characteristics and corresponding definitions, descriptions, and scores as suggested by McNiel et al. (J Am Vet Med Assoc. 1997, 1;211(11):1422-7).

Histologic characteristic	Definition	Description	Score
**Overall differentiation**	Well differentiated	Orderly arrangement of neoplastic cells to other cells, matrix, and basement membrane	1
	Moderately differentiated	Areas with orderly cellular arrangement and areas with loss of cell-to-cell or cell-to-matrix organization	2
	Poorly differentiated	A loss of neoplastic cell orientation to other cells and a loss of polarity to the matrix or basement membrane	3
**Nuclear pleomorphism**	Mild	Overall uniform nuclei with minimal anisocytosis and anisokaryosis	1
	Moderate	Nuclei varied but with less than a two-fold difference in size	2
	Severe	Nuclei with greater than two-fold difference in size and many irregular shapes	3
**Mitoses/ 10 HPF**	1-10	N/A	1
	11-20	N/A	2
	21-30	N/A	3
	> 31	N/A	4
**Nucleolar size**	Small	Difficult to identify	0.5
	Medium	Identifiable but not prominent	1
	Large	Prominent and at least a third of the size of the nucleus	1.5
**Amount of tumor necrosis**	None	N/A	0
	1-20%	N/A	1
	21-50%	N/A	2
	> 51%	N/A	3
**Demarcation of the tumor**	Well demarcated	Sharp border between tumor and normal tissue but no capsule	1
	Moderately demarcated	Areas in which clumps of tumor cells protruded into surrounding normal tissue	2

	Invasive	Many clumps and separate cells protruded into surrounding normal tissue, borders not distinguishable	3

N/A, not applicable.
